# WHAT IT TAKES TO COMBAT DECLINE: LEARNING THROUGH AN INTERPROFESSIONAL STUDENT SENIOR PARTNERSHIP

**DOI:** 10.1093/geroni/igz038.2459

**Published:** 2019-11-08

**Authors:** Anne D Katz, Judy Axonovitz

**Affiliations:** USC Suzanne Dworak-Peck School of Social Work, Los Angeles, California, United States

## Abstract

Healthcare is in need of a workforce with the knowledge and skills to meet the needs of our aging population. Seniors face social, mental, and physical issues as they move into the later stages of life. The Student Senior Partnership Program (SSPP) connects teams of students with healthy senior volunteers in the community. Through this partnership, students learn the strategies seniors implement to stay engaged and maintain their activities of daily living to combat decline and frailty. The SSPP focuses on increasing student’s capability to assess older adults, and learn from their senior within an interprofessional team. Faculty provided training to the senior volunteers so they were prepared to function in a role as “teachers” to students from six professional disciplines (Medicine, Social Work, OT, PT, Dentistry, Pharmacy) through meaningful dialogue focused on physical well-being, relationships with family, friends, community, memory and cognition . Evaluation was performed using the Geriatrics Attitudes Scale (GAS). The pre and post survey (n=72) measured the reported capability to assess older adults and evaluate their attitudes toward the elderly. There were statistically significant improvements in six of nine assessment topics. The largest improvements were in the capability to assess resource gaps (mean=2.79 to 3.44 on a 5-point scale, p<0.001), mental status (3.03 to 3.57, p<0.001) and fall risk (2.99 to 3.50, p<0.001). Attitudes toward the elderly were measured using the Geriatrics Attitudes Scale (GAS). Overall, students improved during the program (mean=3.88 to 4.05 on a 5-point Likert scale, p<0.001).

